# Supplementary material for: Mesolimbic dopamine release precedes actively sought aversive stimuli in mice
Source: Nat Commun. 2023 Apr 27;14:2433. doi: 10.1038/s41467-023-38130-3 (PMC10140067; doi:10.1038/s41467-023-38130-3)
Supplement: Supplementary file 1 — Supplementary Information [file 41467_2023_38130_MOESM1_ESM.pdf]

Supplementary Materials for

Mesolimbic dopamine release precedes actively sought aversive stimuli in mice

Yosuke Yawata, Yu Shikano, Jun Ogasawara, Kenichi Makino, Tetsuhiko Kashima, Keiko Ihara, Airi Yoshimoto, Shota Morikawa, Sho Yagishita, Kenji F. Tanaka, Yuji Ikegaya

Correspondence to: yuji@ikegaya.jp

This PDF file includes:

Fig. S1 – S16

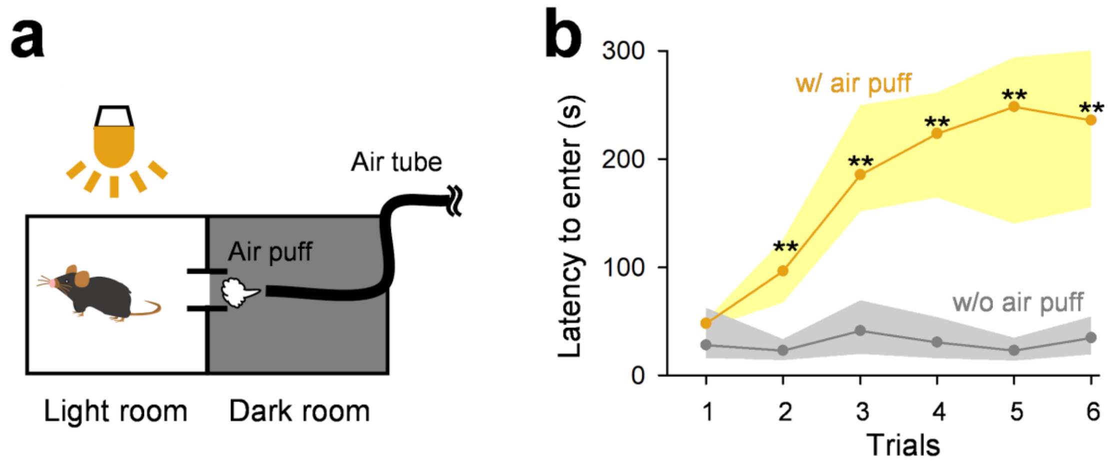

Supplementary Fig. 1. Air-puff stimuli are aversive to mice.
a, Experimental design for a passive avoidance test, in which the latency of a mouse put in a lighted room to enter a dark room was measured for up to 300 s. When the mouse entered the dark room, an air-puff stimulus was immediately applied to the animal's face. The test was repeated 6 times at an interval of 5 min. **b**, Latencies to enter the dark room with (yellow) or without air puffs (dark) for 6 trials. Under the with-air puff conditions, the latency increased over sessions, indicating that mice avoided air puffs. Data represent the means (lines) and 95% CIs (shaded areas) defined by two-sample bootstraps. $^{**}P$ = 0.0050, 0.0040, 0.0012, 0.0012, 0.0022 for the second to sixth trials, respectively. n = 6 mice each, two-sided two-sample bootstrap test.

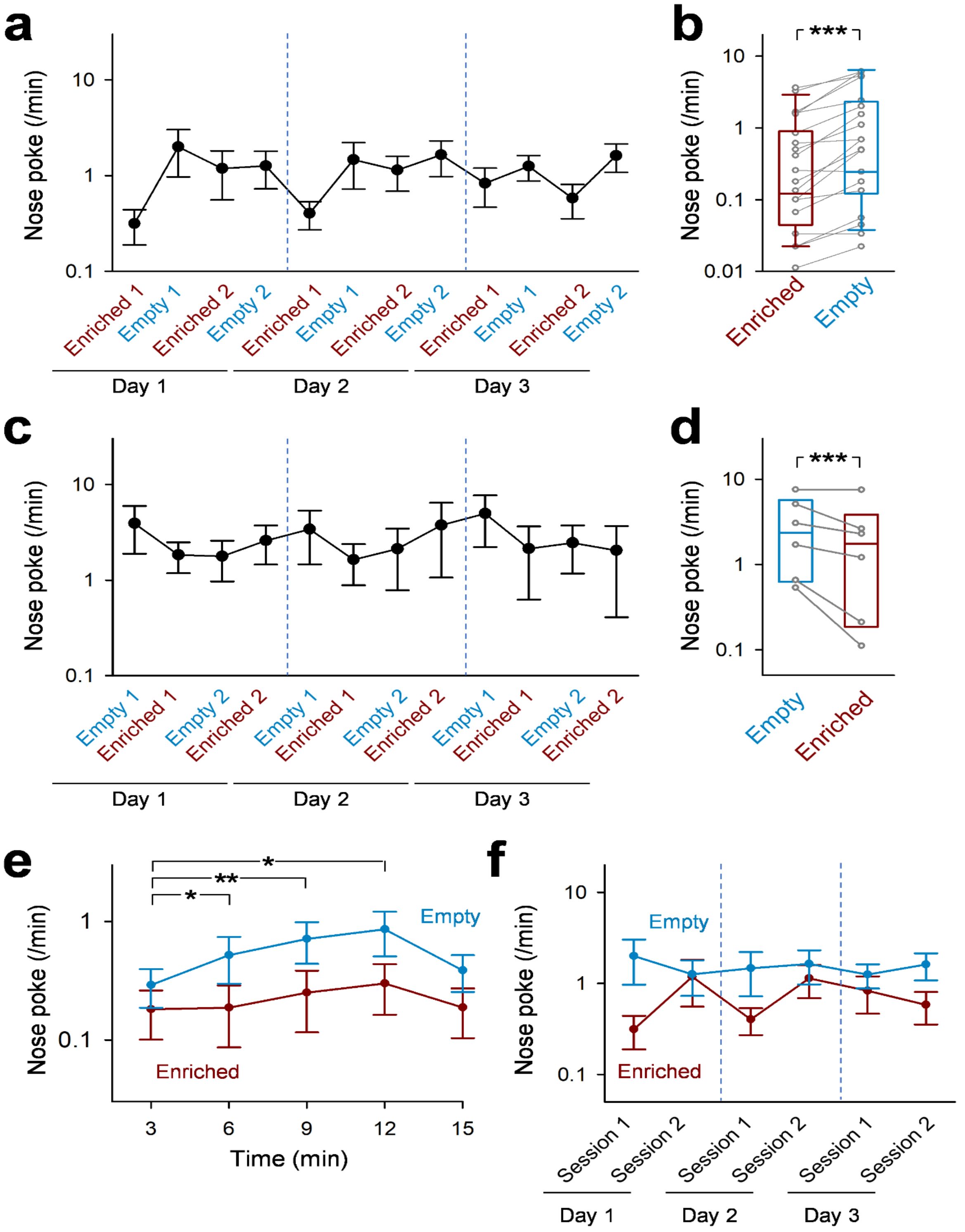

Supplementary Fig. 2. Neither the order of the sessions nor the passage of time affected the nose-poke frequency. a, The time course of the nose-poke frequencies in the test that started with an enriched session. Data are presented as mean values ± SEM from 23 mice. **b**, The mean nose-poke frequency in enriched and empty sessions (same as in Fig. 1c). $^{***}P < 0.001$, $n = 23$ mice, two-sided paired bootstrap test. **c, d**, Same as a and b, but for the test that started with an empty session. Data are presented as mean values ± SEM from 6 mice. $^{***}P < 0.001$, $n = 6$ mice, two-sided paired bootstrap test. **e**, Time evolution of the nose-poke frequency in each session. $^{*}P = 0.011$, 0.014 for 0-3 min versus 3-6 and 9-12 min, respectively. $^{**}P = 0.0024$, $n = 23$ mice, two-sided paired bootstrap test with Bonferroni correction. Data are presented as mean values ± SEM from 23 mice. **f**, The mean nose-poke frequencies over 3 days. Data are presented as mean values ± SEM from 23 mice.

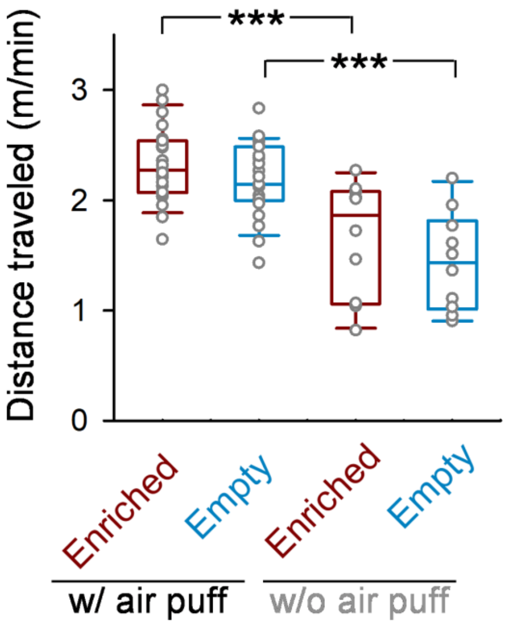

Supplementary Fig. 3. Aversive air puffs did not decrease the exploratory behavior of mice. Distance traveled during the behavioral tests w/and w/o air-puff conditions. The box plots indicate the median, interquartile range, and extreme values. $^{***}P < 0.001$, $n_{\text{w/air puff}} = 23$ mice, $n_{\text{w/o air puff}} = 10$ mice, two-sided two-sample bootstrap test with Bonferroni correction.

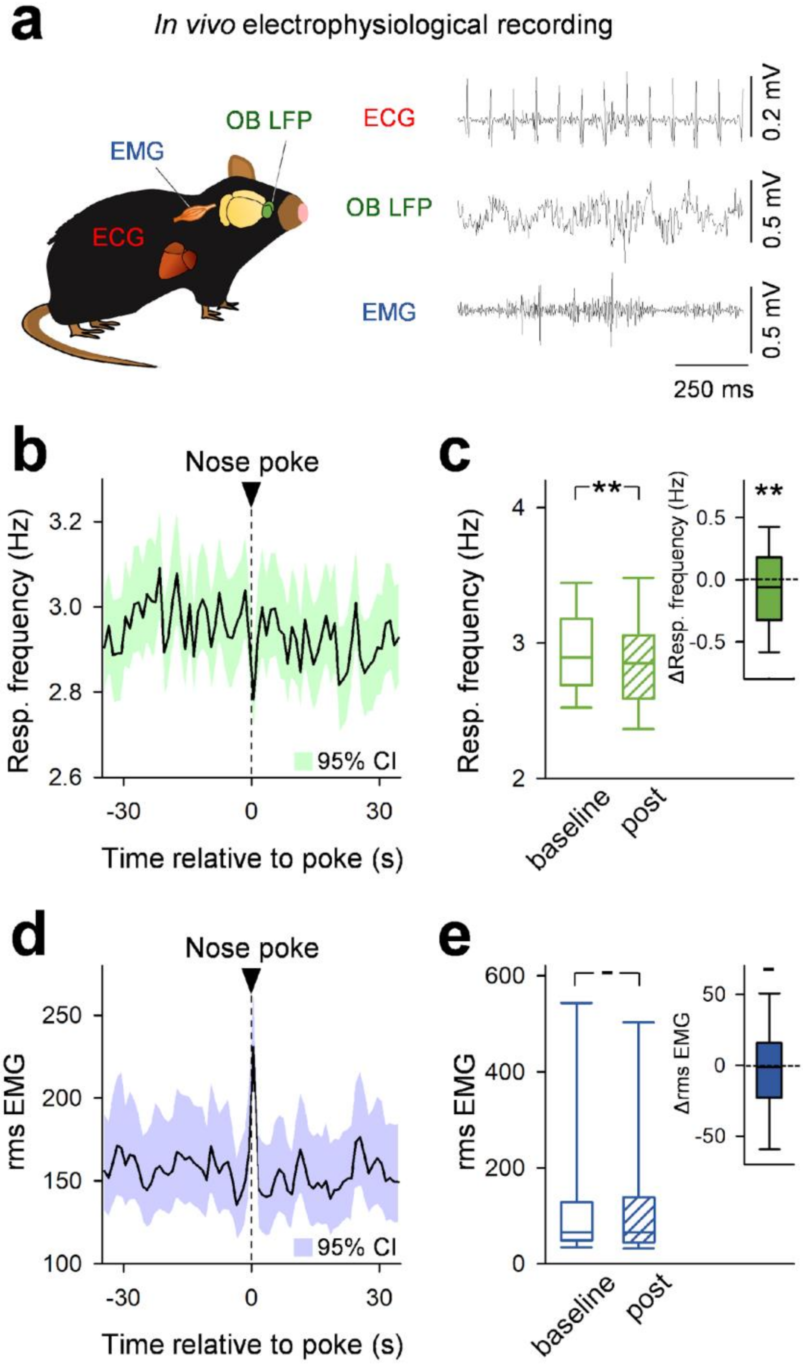

Supplementary Fig. 4. Nose pokes change peripheral signals.
a, *Left*: Schematic illustration of electrophysiological recordings of multiple biosignals and representative traces of 20-200 Hz bandpass-filtered electrocardiograms (ECGs), 6 Hz highpass-filtered olfactory bulb (OB) local field potentials (LFPs), and 100 Hz highpass-filtered dorsal neck muscle electromyograms (EMGs). **b**, Time change in the respiratory rates detected by OB LFPs before and after a nose poke. The line and green areas represent the means and the 95% CIs, respectively, defined by one-sample bootstrapping. n = 263 events. **c**, Mean respiratory rates 30 s before (baseline) and after (post) nose pokes. The inset graph is a plot of the difference between the baseline and post periods. The box plots indicate the median, interquartile range, and extreme values. $^{**}P = 0.0052$, n = 263 events from 4 mice, two-sided paired bootstrap test. **d**, The same as (b) but for the root mean square of EMGs (rmsEMGs). The thick line and blue areas represent the means and the 95% CIs, respectively, calculated by one-sample bootstrapping. n = 294 events. **e**, The same as (c), but for rmsEMG. The box plots indicate the median, interquartile range, and extreme values. $^{-}P = 0.39$, n = 294 events from 6 mice, two-sided paired bootstrap test.

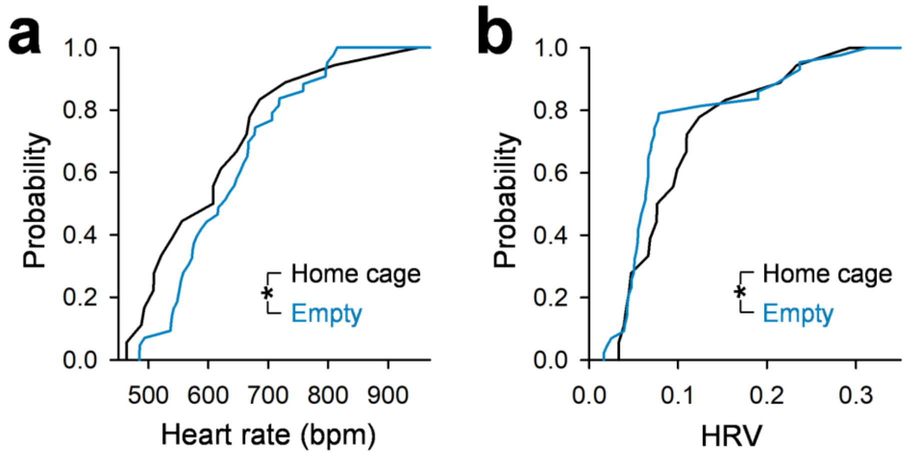

Supplementary Fig. 5. The HRVs in the empty chamber are higher than those in the home cage.

a, Cumulative probabilities of the mean heart rates in the empty chamber and the home cage. $P = 0.032$, $n_{\text{home cage}} = 18$ mice, $n_{\text{empty}} = 43$ mice, two-sided two-sample Kolmogorov–Smirnov test. **b**, Same as a, but for the mean HRVs. $P = 0.026$, $n_{\text{home cage}} = 18$ mice, $n_{\text{empty}} = 43$ mice, two-sided two-sample Kolmogorov–Smirnov test.

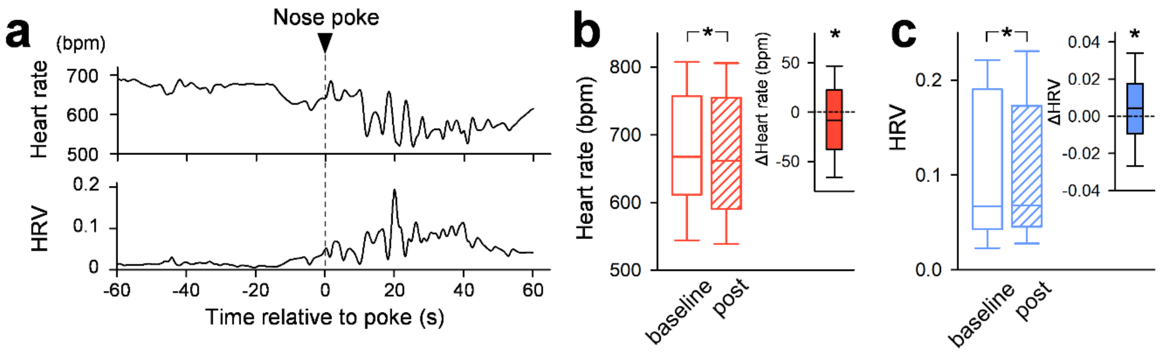

Supplementary Fig. 6. Heart rate decreases after nose poking behavior. a, Representative time course in the heart rates (top) and the heart rate variabilities (HRVs, bottom) aligned to a nose poke. **b**, Mean heart rates 60 s before (baseline) and after (post) nose pokes. The inset graph shows a plot of the difference between the baseline and post periods $^{*}P = 0.017$, $n = 294$ events from 6 mice, two-sided paired bootstrap test. The box plots indicate the median and interquartile range. **c**, The same as (b), but for HRV. The box plots indicate the median and interquartile range. $^{*}P = 0.013$, $n = 294$ events from 6 mice, two-sided paired bootstrap test.

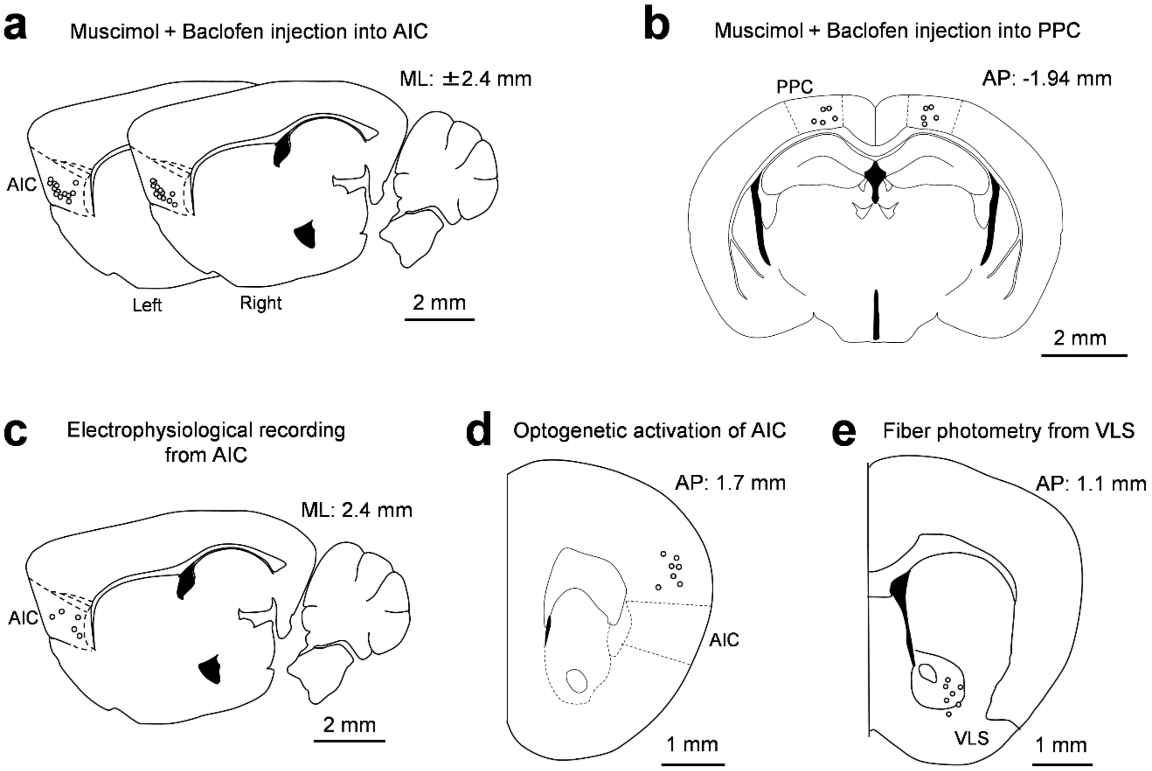

Supplementary Fig. 7. Histological verification of the placement of the tips of drug cannulas, tetrodes, and optical fibers.

a, Brain maps for the identified locations (open circles) of the tips of injection cannulas for Mus+Bac injection into the AIC. *n* = 12 mice. **b**, Same as (a) but for the PPC. *n* = 5 mice. **c**, Brain map for the identified locations of the tips of electrodes for electrophysiological recordings from the AIC. *n* = 5 electrodes from 4 mice. **d**, Schematic visualization of the tips of optic fibers for ChR2 stimulation. *n* = 7 mice. **e**, Same as (d) but for optic fibers for $GRAB_{DA2m}$ imaging. *n* = 8 mice (the tip of the fiber could not be verified for one mouse).

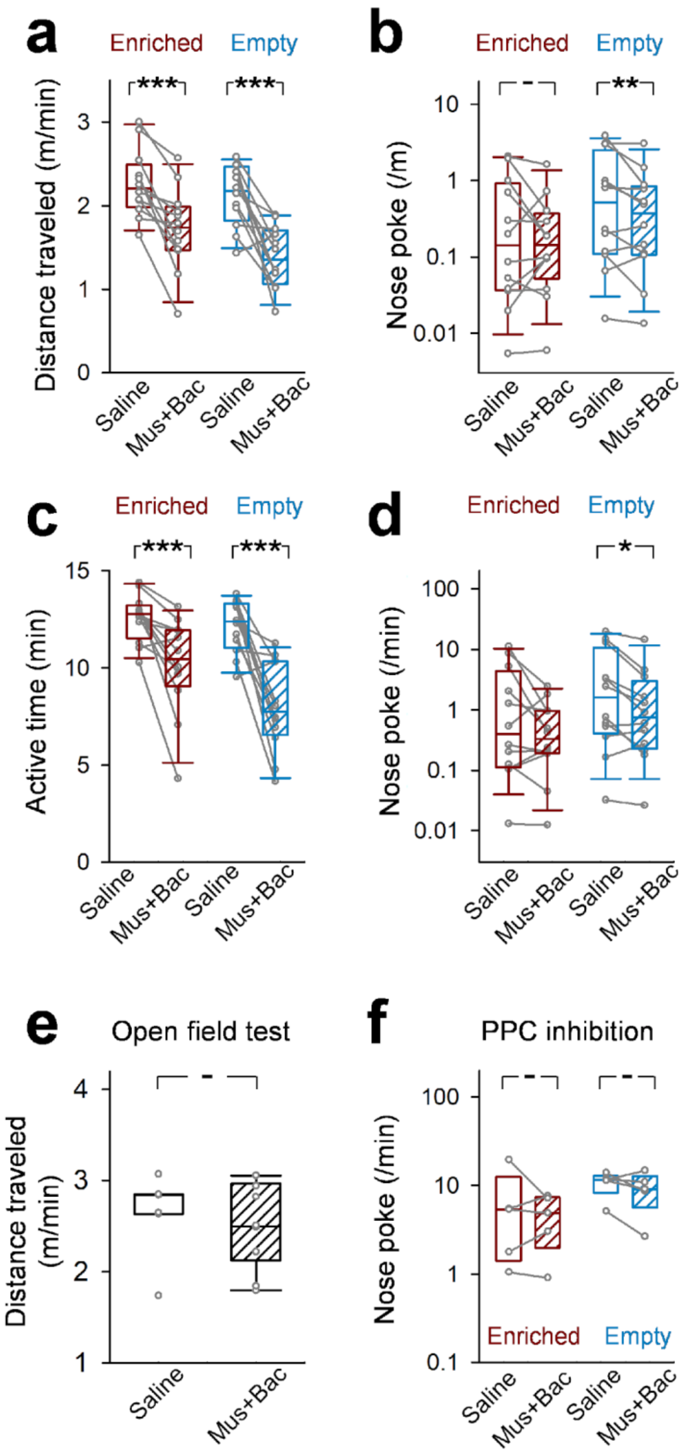

Supplementary Fig. 8. Infusion of GABA receptor agonists into the AIC does not alter locomotor activity in the novel open field test.

a, Distances traveled in the enriched and empty chambers by mice in which the AIC was treated with saline or a mixture of muscimol and baclofen (Mus+Bac). The box plots indicate the median, interquartile range, and extreme values. $^{***}P < 0.0001$, $n = 12$ mice, two-sided paired bootstrap test with Bonferroni correction. **b**, Same as (a) but for the frequencies of nose pokes divided by the distances traveled. The box plots indicate the median, interquartile range, and extreme values. $^{-}P = 0.22$, $^{**}P = 0.008$, $n = 12$ mice, two-sided paired bootstrap test with Bonferroni correction. **c**, Same as (a) but the active times when the running speed was more than 10 cm/s. The box plots indicate the median, interquartile range, and extreme values. $^{***}P < 0.0001$, $n = 12$ mice, two-sided paired bootstrap test with Bonferroni correction. **d**, Same as (a) but for the frequencies of nose pokes divided by the total active time. The box plots indicate the median, interquartile range, and extreme values. $^{*}P = 0.0328$, $n = 12$ mice, two-sided paired bootstrap test with Bonferroni correction. **e**, Distances traveled in the open field test by mice treated with saline and Mus+Bac. The box plots indicate the median, interquartile range, and extreme values. $^{-}P = 0.43$, $n_{\text{Saline}} = 7$ mice, $n_{\text{Mus+Bac}} = 10$ mice, two-sided two-sample bootstrap test. **f**, Same as (a) but for the frequencies of nose pokes with saline or Mus+Bac injection into the posterior parietal cortex (PPC). The box plots indicate the median and interquartile range. $^{-}P > 0.99$, $^{-}P = 0.27$ for the enriched and the empty conditions, respectively. $n = 5$ mice, two-sided paired bootstrap test with Bonferroni correction.

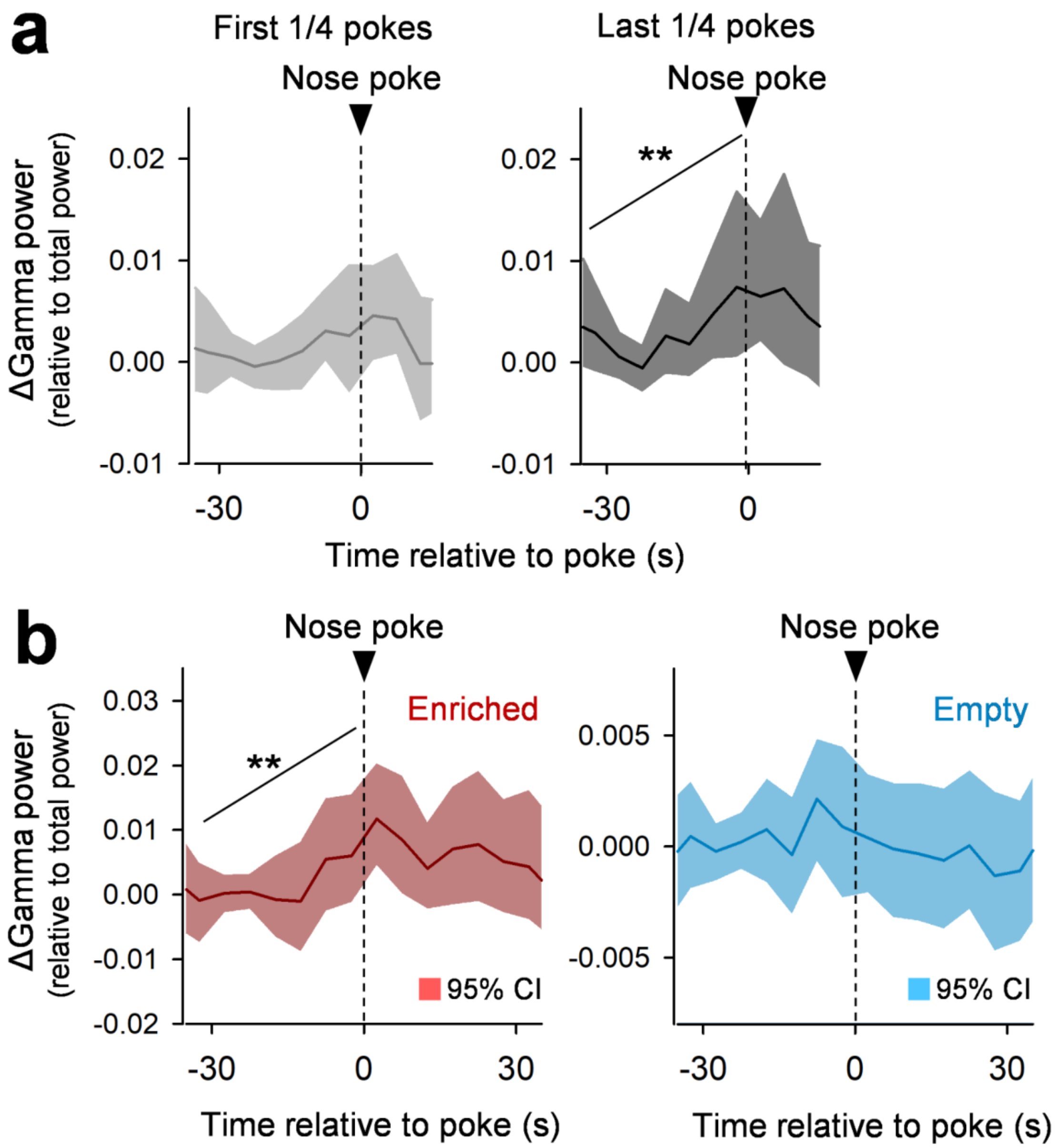

Supplementary Fig. 9. Time evolution of AIC gamma oscillation.
a) Time change in gamma oscillation (30–150 Hz) power around nose pokes in the first quarter (left) and the last quarter (right) of pokes. The thick lines and gray areas represent the means and the 95% confidence intervals (CIs) defined by a one-sample bootstrap test for 18 events, respectively. $^{**}P = 0.0034$, one-sided Jonckheere trend test for the -30-to-0-s period. **b**) Same as (a), but for nose pokes in the enriched (left) and empty (right) sessions. $^{**}P = 0.0079$, one-sided Jonckheere trend test for the -30-to-0-s period. $n_{\mathrm{Enriched}} = 39$ events, $n_{\mathrm{Empty}} = 134$ events.

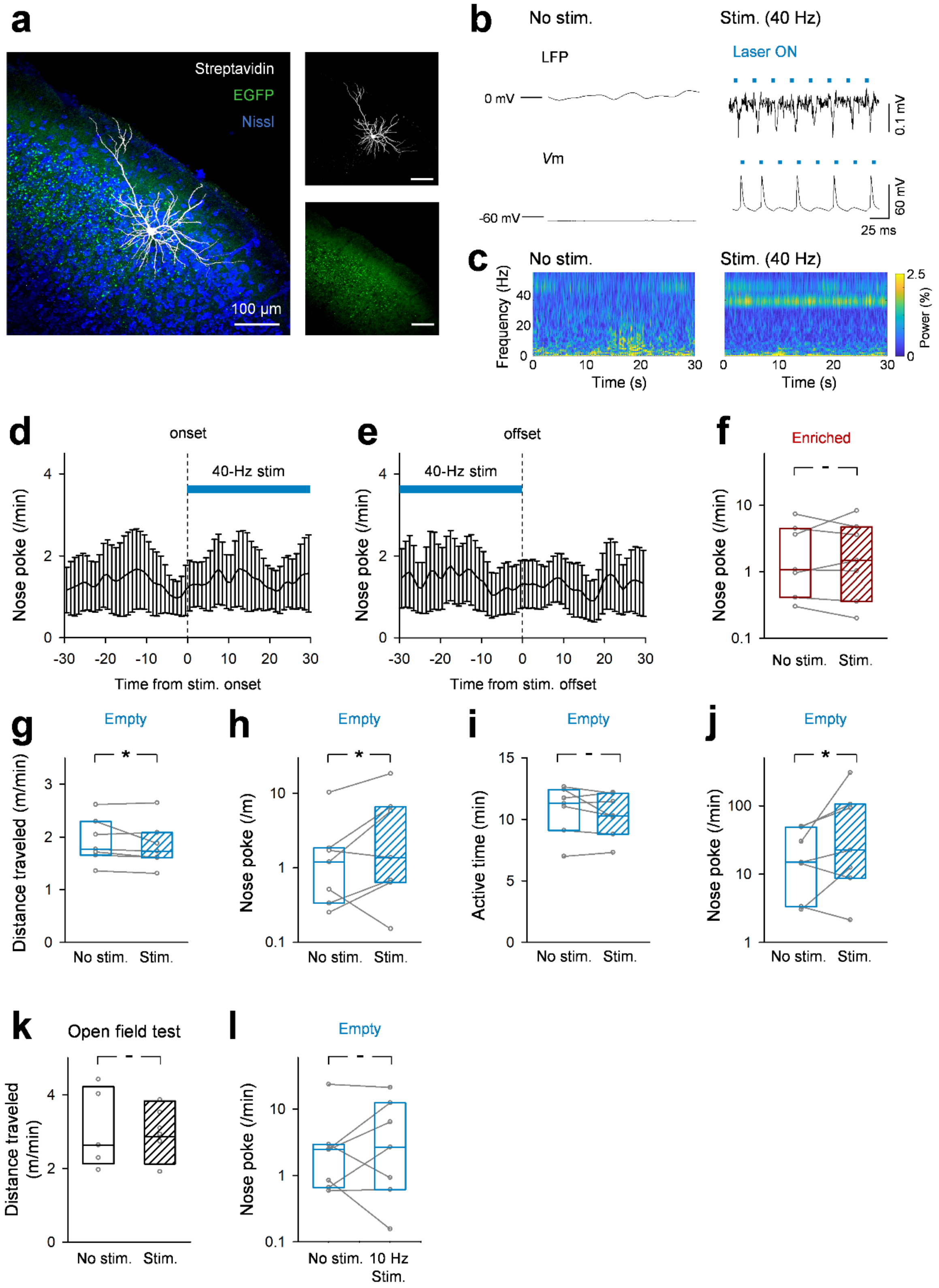

Supplementary Fig. 10. Optogenetic induction of gamma oscillations increases nose-poking behavior.

a, A neuron patch-clamped in a neocortical slice prepared from a mouse that received an injection of AAV-CaMKIIa-ChR2-EYFP into the AIC was confocally visualized with a streptavidin-Alexa Fluor 647 conjugate (white). The slice was counterstained with Nissl. Similar macrographs were obtained from independent experiments performed on 3 mice. **b**, *Top*: Representative AIC LFP traces (top) and intracellular membrane potentials (*V*m) in an AIC pyramidal cell (bottom) without (left) or with 40-Hz blue-light stimulation (right). The blue bars indicate the timings of blue-light laser illumination. **c**, Power spectrograms of the AIC

LFP traces shown in (b). **d,** The frequency of nose pokes time-locked to the 40-Hz blue-light stimulation onset. Data are presented as mean values ± SEM from 7 mice. **e,** Same as (d), but for the 40-Hz blue-light stimulation offset. Data are presented as mean values ± SEM from 7 mice. **f,** The frequencies of nose pokes in an empty room did not increase in mice with blue-light stimulation. The box plots indicate the median and interquartile range. $^{-}P = 0.66$, $n = 7$ mice, two-sided paired bootstrap test. **g**, Distance traveled in an empty room by mice treated without (no stim) and with 40-Hz blue-light stimulation of the AIC (stim). The box plots indicate the median and interquartile range. $^{*}P = 0.018$, $n = 7$ mice, two-sided paired bootstrap test. **h**, Same as (g), but for the frequencies of nose pokes divided by the distance mice traveled. $^{*}P = 0.021$, $n = 7$ mice, two-sided paired bootstrap test. The box plots indicate the median and interquartile range. **i**, Same as (g), but for the active time. The box plots indicate the median and interquartile range. $^{-}P = 0.44$, $n = 7$ mice, two-sided paired bootstrap test with Bonferroni correction. **j**, Same as (g), but for the frequency of nose pokes divided by the active time. The box plots indicate the median and interquartile range. $^{*}P = 0.0368$, $n = 7$ mice, two-sided paired bootstrap test with Bonferroni correction. **k**, Distance mice traveled in the open field test. The box plots indicate the median and interquartile range. $^{-}P = 0.86$, $n_{\text{No stim.}} = 5$ mice, $n_{\text{Stim.}} = 7$ mice, two-sided two-sample bootstrap test. **l**, The frequencies of nose pokes did not increase upon 10-Hz blue-light stimulation. The box plots indicate the median and interquartile range. $^{-}P = 0.11$, $n = 7$ mice, two-sided paired bootstrap test.

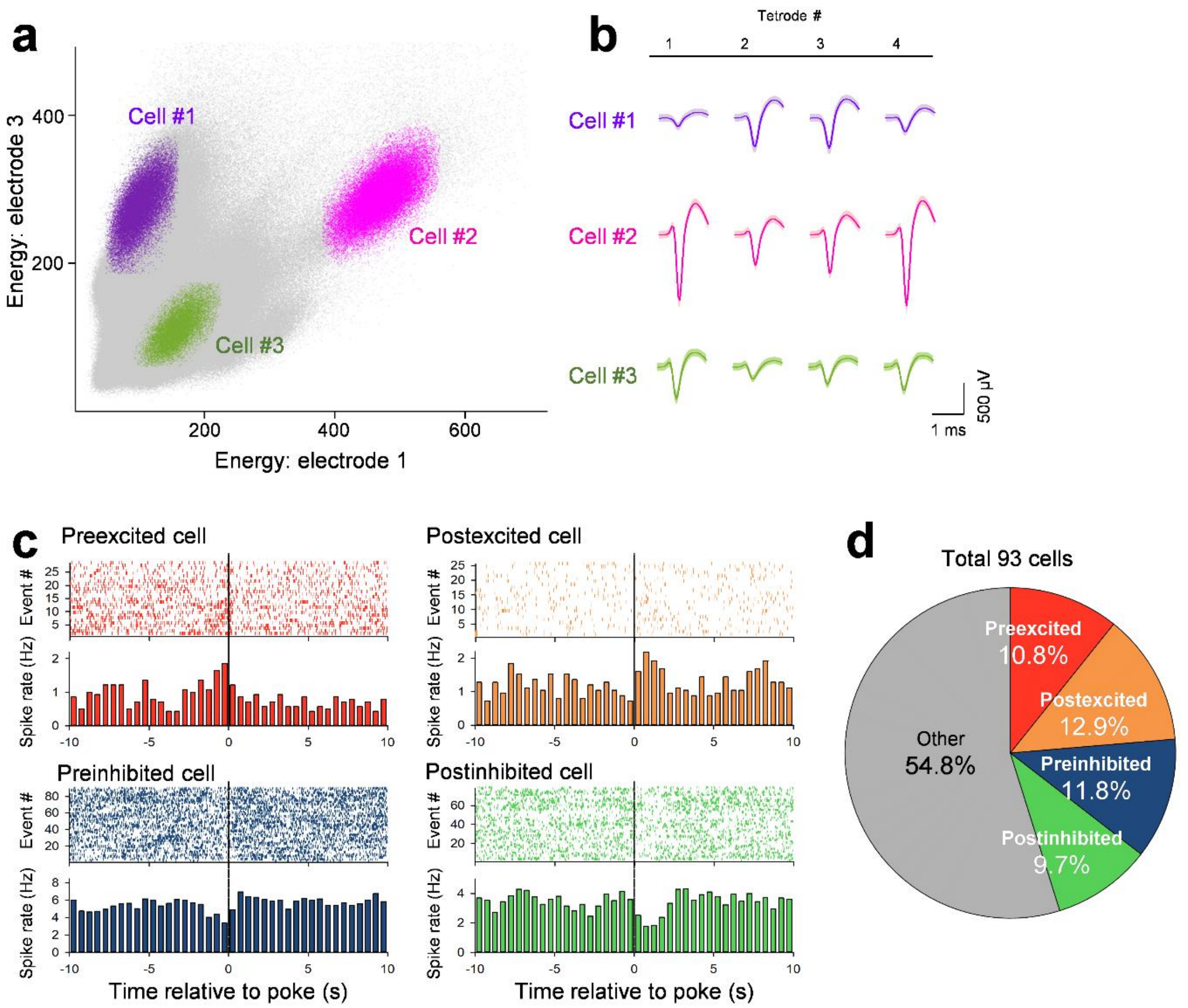

Supplementary Fig. 11. AIC neurons exhibited diverse responses to nose pokes.
a, Spikes were classified using features of their waveforms recorded simultaneously from four electrode sites in a tetrode. Each color represents a cluster of spikes that were estimated to arise from a single neuron. **b**, Representative spike traces recorded from four electrodes. The line and shaded area represent the means and SDs, and their colors correspond to those shown in (a). **c**, Examples of nose poke-responsive cells. *Top left to bottom right*: Data from a cell that significantly increased its firing rate before nose pokes (preexcited cell), a cell that significantly increased its firing rate after nose pokes (postexcited cell), a cell that significantly decreased its firing rate before nose pokes (preinhibited cell), and a cell that significantly decreased its firing rate after nose pokes (postinhibited cell). Significance was determined using a two-sided one-sample bootstrap test ($P < 0.05$). **d**, Distribution of four types of nose poke-responsive cells. $n = 93$ putative excitatory AIC neurons. The simultaneous increase and decrease in firing rates may reflect a fundamental property of neural circuit responses, which probably enhances the signal-to-noise ratio by increasing the contrast of the signal (*e.g.*, via lateral inhibition or balanced excitation/inhibition).

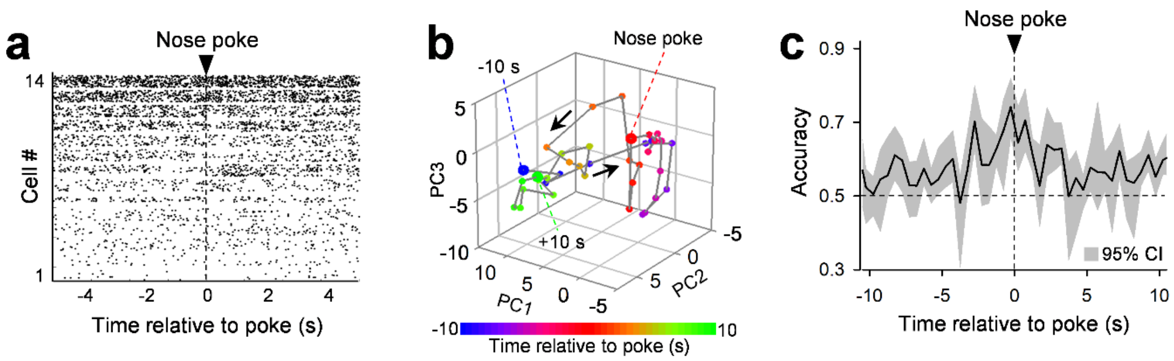

Supplementary Fig. 12. Neuronal activity of the AIC predicts nose-poking behavior. a, Representative raster plot showing the spike patterns of 14 simultaneously recorded AIC neurons. Each neuron contains data from 25 nose pokes. **b**, Representative plot of a spike pattern in the first 3 principal components (PCs). The elapsed time relative to a nose poke is shown on a pseudocolor scale. **c**, Classification accuracy of the firing patterns of the AIC neurons using a support vector machine. The line and gray areas represent the means and the 95% CIs defined by a two-sided one-sample bootstrap test for 7 mice over 8 recording days. The horizontal broken line indicates the chance level.

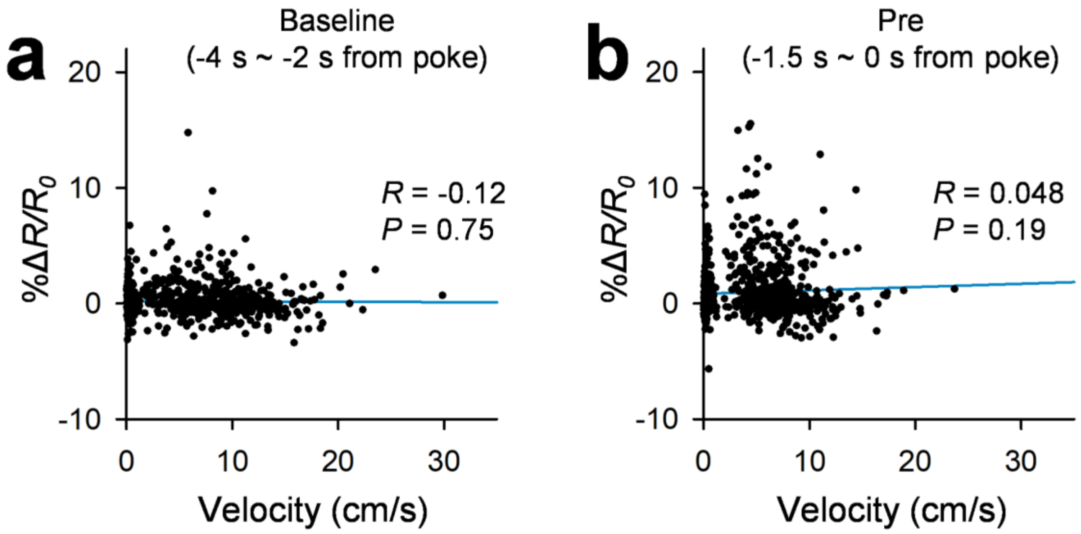

Supplementary Fig. 13. Relationship between velocity before nose poke and dopamine level.

a, The relationship between the velocity during the baseline period (-4 s to -2 s relative to nose pokes) and the AUC of $\Delta R/R_0$ during the same period. Pearson correlation coefficient R = -0.12, P = 0.75, n = 761 events from 8 mice. **b**, Same as (a), but for the prepoke period (-1.5 s to 0 s relative to nose pokes). R = 0.048, P = 0.19, n = 761 events from 8 mice. Pearson correlation coefficient.

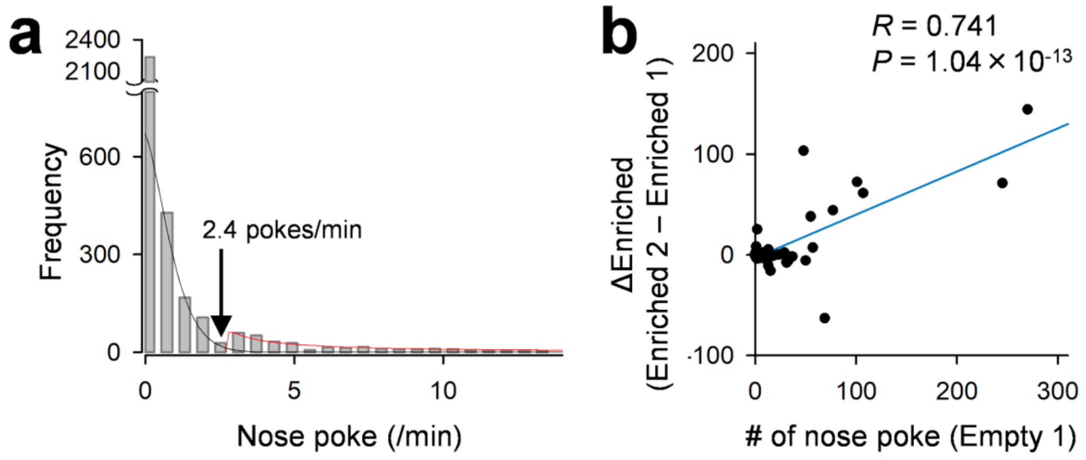

Supplementary Fig. 14. Distributions of the nose-poke frequencies indicate intense behavioral states in mice.

a, Histogram of the frequencies of nose pokes in a total of 55 mice. The nose-poke frequency approximated a distribution consisting of a Poisson distribution (black) and a log-normal distribution (red). The frequency of nose pokes exhibited a local minimum at 2.4 pokes/min, which was used to detect the onset of intense behavioral states. **b**, The relationship between the number of nose pokes in a given empty session (Empty 1 shown in Fig. 1b) and the increase in the numbers of nose pokes between two successive enriched sessions (the difference between Enriched 1 and Enriched 2). $R = 0.741$, $P = 1.04 \times 10^{-13}$, $n = 55$ sessions, Pearson correlation coefficient.

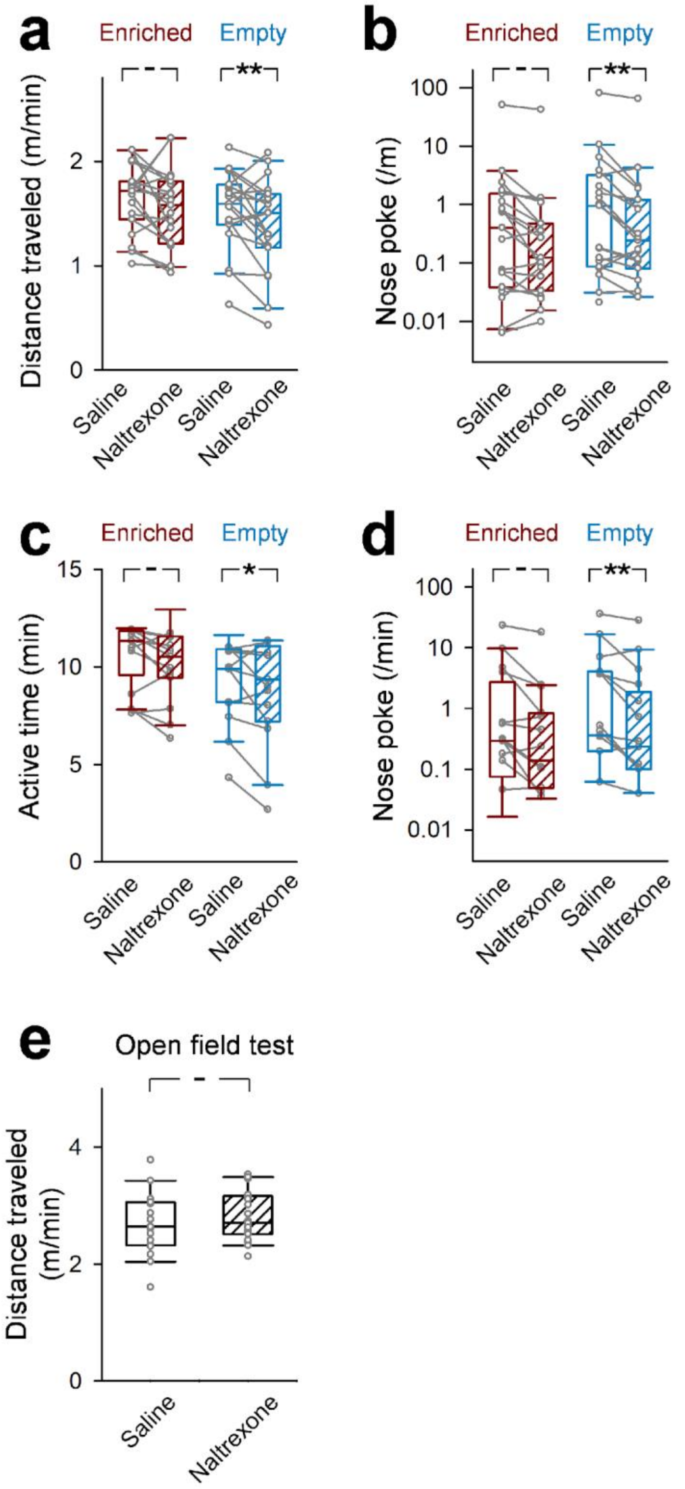

Supplementary Fig. 15. Naltrexone administration does not alter locomotor activity.

a, Distances traveled in the enriched or empty chambers by mice treated intraperitoneally with saline or with 10 mg/kg naltrexone. The box plots indicate the median, interquartile range, and extreme values. $^{**}P = 0.0064$, $n = 19$ mice, two-sided paired bootstrap test with Bonferroni correction. **b**, Same as (a) but for the frequencies of nose pokes divided by the distances mice traveled. The box plots indicate the median, interquartile range, and extreme values. $^{**}P = 0.0052$, $n = 19$ mice, two-sided paired bootstrap test with Bonferroni correction. **c**, Same as (a) but for the active times. The box plots indicate the median, interquartile range, and extreme values. $^{*}P = 0.017$, $n = 19$ mice, two-sided paired bootstrap test with Bonferroni correction. **d**, Same as (a) but for the frequencies of nose pokes divided by the active times. The box plots indicate the median, interquartile range, and extreme values. $^{**}P = 0.0068$, $n = 19$ mice, two-sided paired bootstrap test with Bonferroni correction. **e**, Distances traveled in the open field test by mice treated with saline and with naltrexone injection. The box plots indicate the median, interquartile range, and extreme values. $^{-}P = 0.82$, $n_{\text{Saline}} = 9$ mice, $n_{\text{Naltrexone}} = 10$ mice, two-sided two-sample bootstrap test.

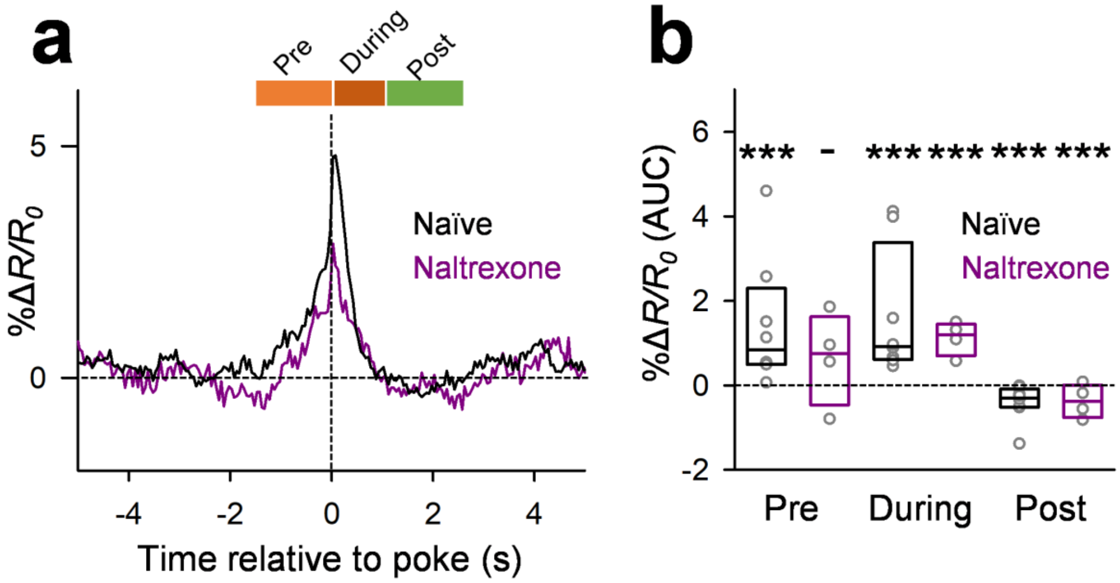

Supplementary Fig. 16. Naltrexone administration decreases dopamine release in the VLS before nose pokes. a, Mean $\Delta R/R_0$ of $GRAB_{DA2m}$ signals from naïve mice (black) and mice treated with 10 mg/kg naltrexone (purple). **b**, AUC of $\Delta R/R_0$ during the prepoke period (left), the during-poke period (middle), and the postpoke period (right). The black and purple box plots indicate the naïve and naltrexone conditions, respectively. The box plots indicate the median and interquartile range. $^{-}P = 0.25$, $^{***}P < 0.0001$, $n_{\mathrm{Naïve}} = 6$ mice, $n_{\mathrm{Naltrexone}} = 4$ mice, two-sided one-sample bootstrap test.
