# Supplementary material for: Mesolimbic dopamine release precedes actively sought aversive stimuli in mice
Source: Nat Commun. 2023 Apr 27;14:2433. doi: 10.1038/s41467-023-38130-3 (PMC10140067; doi:10.1038/s41467-023-38130-3)
Supplement: Supplementary file 3 — Description of Additional Supplementary Files [file 41467_2023_38130_MOESM3_ESM.pdf]

File name: Supplementary Movie 1

Description: Mouse in enriched and empty chambers A mouse in a chamber enriched with toys rarely poked its nose into a hole that triggered air puffs onto its face, whereas the same mouse frequently exhibited nose pokes when placed in an empty chamber. Video plays at 1× speed.

File name: Supplementary Movie 2

Description: Mouse in an intense behavioral state A mouse that developed intense behavioral states repeatedly sought air puffs. Video plays at double speed.
