# Supplementary material for: Mesolimbic dopamine release precedes actively sought aversive stimuli in mice
Source: Nat Commun. 2023 Apr 27;14:2433. doi: 10.1038/s41467-023-38130-3 (PMC10140067; doi:10.1038/s41467-023-38130-3)
Supplement: Supplementary file 6 — Reporting Summary [file 41467_2023_38130_MOESM6_ESM.pdf]

nature portfolio

Corresponding author(s): Yuji Ikegaya

Last updated by author(s): Apr 3, 2023

Reporting Summary

Nature Portfolio wishes to improve the reproducibility of the work that we publish. This form provides structure for consistency and transparency in reporting. For further information on Nature Portfolio policies, see our Editorial Policies and the Editorial Policy Checklist.

Statistics

For all statistical analyses, confirm that the following items are present in the figure legend, table legend, main text, or Methods section.

n/a	Confirmed	
☐	☒	The exact sample size (*n*) for each experimental group/condition, given as a discrete number and unit of measurement
☐	☒	A statement on whether measurements were taken from distinct samples or whether the same sample was measured repeatedly
☐	☒	The statistical test(s) used AND whether they are one- or two-sided<br>*Only common tests should be described solely by name; describe more complex techniques in the Methods section.*
☒	☐	A description of all covariates tested
☐	☒	A description of any assumptions or corrections, such as tests of normality and adjustment for multiple comparisons
☐	☒	A full description of the statistical parameters including central tendency (e.g. means) or other basic estimates (e.g. regression coefficient) AND variation (e.g. standard deviation) or associated estimates of uncertainty (e.g. confidence intervals)
☐	☒	For null hypothesis testing, the test statistic (e.g. *F*, *t*, *r*) with confidence intervals, effect sizes, degrees of freedom and *P* value noted<br>*Give P values as exact values whenever suitable.*
☒	☐	For Bayesian analysis, information on the choice of priors and Markov chain Monte Carlo settings
☒	☐	For hierarchical and complex designs, identification of the appropriate level for tests and full reporting of outcomes
☐	☒	Estimates of effect sizes (e.g. Cohen's *d*, Pearson's *r*), indicating how they were calculated

Our web collection on statistics for biologists contains articles on many of the points above.

Software and code

Policy information about availability of computer code

Data collection	Each data collection procedures are described in the Methods. Behavioral data were collected with custom-written scripts in Python 2/3. In vivo electrophysiological signals were recorded by Cereplex Direct recording system (Blackrock Microsystems). In vitro elecrtophysiological signals were amplified and digitized at a sampling rate of 20 kHz using a MultiClamp 700B amplifier and a Digidata 1440A digitizer that was controlled by pCLAMP 10.3 software (Molecular Devices). The fluorescence signals in addition to TTL signals from behavioral settings were digitized by a data acquisition module (cDAQ-9178, National Instruments), and simultaneously recorded using a custom-made LabVIEW program (National Instruments). Any data collection programs are available by sending a request to the corresponding author.
Data analysis	Offline computational analyses were performed using MATLAB (R2022a) and Python 2/3. Each analysis procedure is described in necessary detail within the "Data analysis" section of the Methods. In the description of analysis methods, more<br>detail is provided for novel analysis methods. Any analysis codes are available by sending a request to the corresponding author.

For manuscripts utilizing custom algorithms or software that are central to the research but not yet described in published literature, software must be made available to editors and reviewers. We strongly encourage code deposition in a community repository (e.g. GitHub). See the Nature Portfolio guidelines for submitting code & software for further information.

Data

Policy information about availability of data

All manuscripts must include a data availability statement. This statement should provide the following information, where applicable:

- Accession codes, unique identifiers, or web links for publicly available datasets
- A description of any restrictions on data availability
- For clinical datasets or third party data, please ensure that the statement adheres to our policy

The data of this study are available from the corresponding author upon reasonable request.

Human research participants

Policy information about studies involving human research participants and Sex and Gender in Research.

Reporting on sex and gender	*Use the terms sex (biological attribute) and gender (shaped by social and cultural circumstances) carefully in order to avoid confusing both terms. Indicate if findings apply to only one sex or gender; describe whether sex and gender were considered in study design whether sex and/or gender was determined based on self-reporting or assigned and methods used. Provide in the source data disaggregated sex and gender data where this information has been collected, and consent has been obtained for sharing of individual-level data; provide overall numbers in this Reporting Summary. Please state if this information has not been collected. Report sex- and gender-based analyses where performed, justify reasons for lack of sex- and gender-based analysis.*
Population characteristics	*Describe the covariate-relevant population characteristics of the human research participants (e.g. age, genotypic information, past and current diagnosis and treatment categories). If you filled out the behavioural & social sciences study design questions and have nothing to add here, write "See above."*
Recruitment	*Describe how participants were recruited. Outline any potential self-selection bias or other biases that may be present and how these are likely to impact results.*
Ethics oversight	*Identify the organization(s) that approved the study protocol.*

Note that full information on the approval of the study protocol must also be provided in the manuscript.

Field-specific reporting

Please select the one below that is the best fit for your research. If you are not sure, read the appropriate sections before making your selection.

☒ Life sciences ☐ Behavioural & social sciences ☐ Ecological, evolutionary & environmental sciences

For a reference copy of the document with all sections, see nature.com/documents/nr-reporting-summary-flat.pdf

Life sciences study design

All studies must disclose on these points even when the disclosure is negative.

Sample size	Sample sizes were not predetermined, but our sample sizes are similar to (n animals) or higher than (n traces) those generally employed in the field 21,23,25,37,74,76,79,80,81.
Data exclusions	When optic fiber position, electrode position, or microinjection canula position was not targeted correctly, we excluded those mice.
Replication	We observed similar results which satisfied the same statistical criteria across experiments and we could replicate all our results.
Randomization	All animals and samples were randomly assigned to the experimental groups.
Blinding	Data collection and analysis were not performed blind to the conditions of the experiments.

Reporting for specific materials, systems and methods

We require information from authors about some types of materials, experimental systems and methods used in many studies. Here, indicate whether each material, system or method listed is relevant to your study. If you are not sure if a list item applies to your research, read the appropriate section before selecting a response.

Materials & experimental systems

n/a	Involved in the study	
☐	☒	Antibodies
☒	☐	Eukaryotic cell lines
☒	☐	Palaeontology and archaeology
☐	☒	Animals and other organisms
☒	☐	Clinical data
☒	☐	Dual use research of concern

Methods

n/a	Involved in the study	
☒	☐	ChIP-seq
☒	☐	Flow cytometry
☒	☐	MRI-based neuroimaging

Antibodies

Antibodies used	The primary antibodies used was: anti-GFP (1:500, ab13970; abcam). The secondary antibodies used was: goat anti-chicken-Alexa488 (1:500; A11039; Thermo Fisher Scientific).
Validation	Primary and Secondary antibodies have been validated for use in the system under study in previous publications.<br>Noguchi, A., Huszár, R., Morikawa, S., Buzsáki, G. & Ikegaya, Y. Inhibition allocates spikes during hippocampal ripples. Nat. Commun. 13, 1280 (2022)<br>Iwasaki, S. & Ikegaya, Y. Contextual Fear Memory Retrieval Is Vulnerable to Hippocampal Noise. Cereb. Cortex 31, 785–794 (2021)

Animals and other research organisms

Policy information about studies involving animals; ARRIVE guidelines recommended for reporting animal research, and Sex and Gender in Research

Laboratory animals	In vivo experiments were performed using seven-week-old or older male C57BL/6JJmsSlc mice or C57BL/6NCrSlc mice (SLC, Hamamatsu, Shizuoka, Japan). In vitro experiments were performed using P12 male C57BL/6JJmsSlc mice purchased from SLC (Hamamatsu, Shizuoka, Japan).
Wild animals	No wild animals were used
Reporting on sex	All data were collected in male mice because of the possibility of behavioral variation in female mice.
Field-collected samples	No field-collected samples were used in this study.
Ethics oversight	Animal experiments were performed with the approval of the Animal Experiment Ethics Committee at The University of Tokyo and Keio University School of Medicine.

Note that full information on the approval of the study protocol must also be provided in the manuscript.
